# The chemical component of the mixed GF-TTMn synapse in *Drosophila melanogaster* uses acetylcholine as its neurotransmitter

**DOI:** 10.1111/j.1460-9568.2007.05686.x

**Published:** 2007-07-01

**Authors:** Marcus J Allen, R K Murphey

**Affiliations:** 1 Department of Biosciences, University of Kent, Canterbury Kent, CT2 7NJ, UK; 2 Department of Biology, Morrill Science Center, University of Massachusetts, Amherst Massachusetts 01003, USA

**Keywords:** giant fibre, innexins, neuron, neurotransmitter, tetanus toxin

## Abstract

The largest central synapse in adult *Drosophila* is a mixed electro-chemical synapse whose gap junctions require the product of the *shaking-B (shak-B)* gene. *Shak-B*^2^ mutant flies lack gap junctions at this synapse, which is between the giant fibre (GF) and the tergotrochanteral motor neuron (TTMn), but it still exhibits a long latency response upon GF stimulation. We have targeted the expression of the light chain of tetanus toxin to the GF, to block chemical transmission, in *shak-B*^2^ flies. The long latency response in the tergotrochanteral muscle (TTM) was abolished indicating that the chemical component of the synapse mediates this response. Attenuation of GAL4-mediated labelling by a cha-GAL80 transgene, reveals the GF to be cholinergic. We have used a temperature-sensitive allele of the *choline acetyltransferase* gene (*cha*^ts2^) to block cholinergic synapses in adult flies and this also abolished the long latency response in *shak-B*^2^ flies. Taken together the data provide evidence that both components of this mixed synapse are functional and that the chemical neurotransmitter between the GF and the TTMn is acetylcholine. Our findings show that the two components of this synapse can be separated to allow further studies into the mechanisms by which mixed synapses are built and function.

## Introduction

**Fig. 1:**
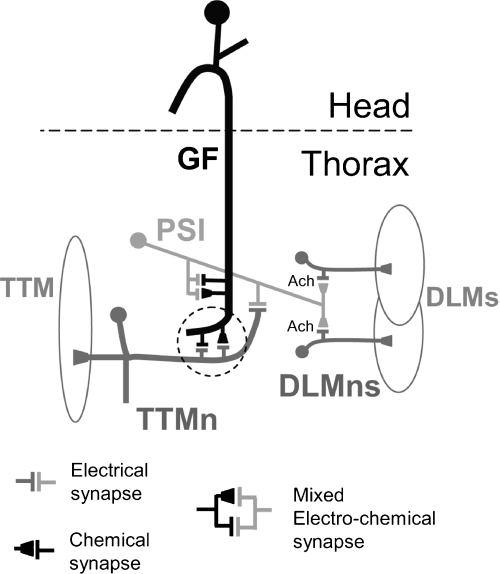
Schematic representing the known synaptic connections of the GFS. For simplicity only one side of the bilateral circuit is shown. The GF makes mixed electrochemical synapses with the PSI and with the TTMn in the thoracic ganglia. The GF-TTMn synapse is circled with a dotted line. The PSI synapses with the DLMns via cholinergic chemical synapses. The PSI synapses with five DLMns, but only two are indicated for clarity. PSI and TTMn are also electrically coupled. Adapted from Allen *et al*. (2006).

Mixed electro-chemical synapses are found in both vertebrate and invertebrate nervous systems including fish (Lin & Faber, 1988; Korn & Faber, 2005), crustaceans (Edwards *et al*., 1999), and insects (Blagburn *et al*., 1999).Their bi-partite nature has traditionally made them difficult to study as the two components are not easily separable. The giant fibre system (GFS) of *Drosophila melanogaster* is a simple neural circuit that mediates an escape response in adult flies and contains mixed electro-chemical synapses (reviewed in Allen *et al*., 2006). The two large giant fibres (GFs) (Fig. 1) relay information from the brain to the thoracic ganglia where they make electro-chemical synapses with the tergotrochanteral motor neuron (TTMn), which drives the leg extensor muscle and the peripherally synapsing interneuron (PSI; King & Wyman, 1980; Blagburn *et al*., 1999), which drives the dorsal longitudinal motor neurons (DLMns; King & Wyman, 1980; Gorczyca & Hall, 1984).

The *shaking-B*^2^ (*shak-B*^2^) mutation was originally generated during an adult EMS behavioural screen (Homyk *et al*., 1980) and independently, *Passover* alleles at the same locus were isolated in a mutagenic screen for flies that failed to escape to a light-off stimulus (Thomas & Wyman, 1984; Baird *et al*., 1990). The mutants show a very specific electrophysiological phenotype upon GF stimulation; a long latency and labile response is seen in tergotrochanteral muscle (TTM) and no responses are elicited in the dorsal longitudinal muscles (DLMs; Thomas & Wyman, 1984; Baird *et al*., 1990). The long latency response in TTM was originally thought to be due either, to a separate pathway from the brain to the thorax that was uncovered once the GF-TTMn synapse was rendered nonfunctional, or to a defect in the GF-TTMn synapse (Thomas & Wyman, 1984; Baird *et al*., 1990). Electrophysiological tests on flies in which neurite outgrowth of the GF was blocked showed that the only pathway from the brain to TTMn is via the GF (Allen *et al*., 2000). This suggested that a defect in the GF-TTMn synapse was the cause of the long latency seen in TTM. Moreover, it was shown that *shak-B*^2^ encodes a gap junction protein and the mutant flies have no functional gap junctions between the GF and TTMn (Phelan *et al*., 1996; Sun & Wyman, 1996; Phelan *et al*., 1998). EM work has revealed the existence of T-bars and synaptic vesicles, indicative of chemical transmission, in the presynaptic bends of the GF and at the GF-PSI contact points (Blagburn *et al*., 1999). This body of evidence led to the hypothesis that the chemical component of the synapse is responsible for the long latency TTM response in *shak-B*^2^ flies.

We have used these *shak-B*^2^ mutants, in combination with misexpression of a toxin, to test the function of the chemical component of this mixed synapse. We provide evidence that the GF is cholinergic by using the expression of GAL80 to block GAL4-mediated labelling of the neuron and have used a temperature sensitive allele that affects acetylcholine (ACh) production to demonstrate the nature of chemical transmission at the synapse.

## Materials and methods

### Drosophila *stocks*

All stocks were cultured at 25 °C on standard medium unless stated otherwise. The P[GAL4] line c17 expresses in the GF and other neurons in the brain and optic lobes as well as sensory neurons. However, it does not express in the TTMn or any other identified neurons within the GFS (Allen *et al*., 1999; Trimarchi *et al*., 1999). The P[GAL4] line A307 expresses in the GF and weakly in the TTMn and some DLMns as well as some other neurons in the CNS (Phelan *et al*., 1996; Allen *et al*., 1998). The UAS-IMPTNT and UAS-TNT(G) lines are described in Sweeney *et al*. (1995) and *Cha*^*3.3kb*^-GAL80 has been described previously (Kitamoto, 2002). The *shaking-B*^2^ (*shak-B*^2^) mutation is an EMS-induced allele from a behavioural screen performed by Homyk *et al*. (1980). It acts as a functional null for the shak-B (neural) and shak-B (neural +16) gene products (Krishnan *et al*., 1993; Krishnan *et al*., 1995; Zhang *et al*., 1999). The *cha*^ts2^ allele used is that originally described by Greenspan *et al*. (1980) and causes an arginine to histidine change at amino acid 397 in the resulting protein (Wang *et al*., 1999). The *CyO* and *MKRS* balancer chromosomes are described in Lindsley & Zimm (1992).

### Electrophysiology of flies expressing the tetanus toxin light chain

Flies were anaesthetized by cooling on ice and waxed onto a small podium, ventral side down, with the wings held outwards and secured in the wax. Tungsten electrodes were pushed through the eyes and into the brain for stimulation and a tungsten ground wire placed into the abdomen. A pulse of 40–60 V for 0.03 ms from a Grass S48 stimulator (Astro-Medical, West Warwick, USA) via a stimulus isolation unit was given to activate the GFs in the brain and recordings were made from the TTM and a contralateral DLM muscle with glass microelectrodes (resistance 40–60 MΩ). These were filled with 3M KCl, or saline and placed into the muscles through the cuticle. Responses were amplified using Getting 5 A amplifiers (Getting Instruments, San Diego, USA) and data digitized using an analogue-digital Digidata 1320 and Axoscope 9.0 software (MDS Inc, Toronto, Canada). For response latency recordings five single stimuli were given to each individual tested with a 5-s rest period between each stimulus. This usually enabled sufficient time for the weak GF-TTMn synapse of *shak-B*^2^ mutant flies to recover. In a few cases, where five responses were not initially obtained, more stimuli were given. To obtain data for synaptic following at two frequencies, trains of ten stimuli, at either 250 Hz or 100 Hz, were given with a 5-s rest period between each train.

For the thoracic stimulation, to activate the motor neurons directly, the stimulating electrodes were moved from the brain and carefully placed through the cuticle at the anterior end of the thorax and down into the fused thoracic ganglia in the ventral part of the thorax.

### Electrophysiology of flies containing cha^ts2^

All *cha*^ts2^ flies were reared at the permissive temperature of 18 °C to allow them to develop to adulthood. Flies were collected on the day they eclosed and either kept at 18 °C for 48 h prior to testing or were moved to an incubating water bath and kept at 28 °C for 48 h prior to testing. Following this flies were prepared for electrophysiology as described above and tested within 10 min of being removed from the 18 °C or 25 °C environment. Each individual was given five stimuli at 1 Hz in the brain and recordings made. At this stimulation rate wild-type flies will show responses in TTM and DLM to every stimulus and *shak-B*^2^ mutants will show no responses in DLM and intermittent responses in TTM (Thomas & Wyman, 1984; Baird *et al*., 1990; this study). The stimulating electrodes were then moved as described above and thoracic stimulation of five stimuli at 1 Hz was given to the same individual.

### CNS histochemistry

Adult nervous systems were dissected in 0.1 m PBS plus 0.1% Triton X-100, fixed briefly in 1% gluteraldehyde and stained for β-galactosidase activity as previously described (Jacobs *et al*., 2000). Images were taken on a Leica DMR microscope and figures assembled using Adobe Photoshop.

## Results

### Blocking chemical synaptic transmission with tetanus toxin

**Fig. 2:**
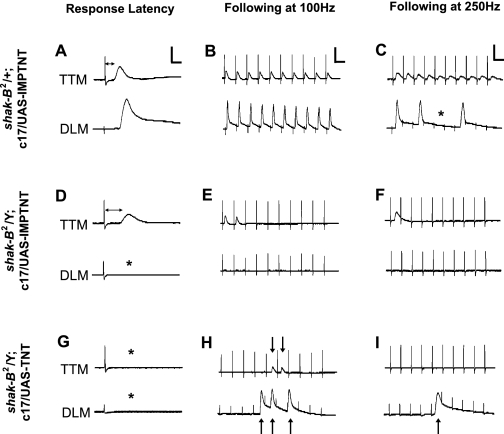
Expression of tetanus toxin in the GF abolishes the TTM response in *shak-B*^2^ mutants. Responses in the TTM and a DLM are shown when individual flies were given a single brain stimulus or ten brain stimuli at either 100 or 250 Hz. (A–C) responses in a *shak-B*^2^/+ c17/UAS-IMPTNT control fly show wild-type latencies and following frequencies at 100 and 250 Hz including the DLM not following 1:1 at 250 Hz (*) due to the failure of the PSI-DLMns synapses (Tanouye & Wyman, 1980). (D–F) responses in a *shak-B*^2^/Y c17/UAS-IMPTNT fly showing no output to DLM and a long latency response and poor following in TTM at both frequencies. (G–I) responses in a *shak-B*^2^/Y c17/UAS-TNT fly show no responses in either TTM or DLM upon stimulation but increased spontaneous activity (marked with arrows). Vertical scale bars, 50 mV for all traces; horizontal, 1 ms for response latencies, 10 ms for following at 100 Hz, and 4 ms for following at 250 Hz.

**Fig. 3:**
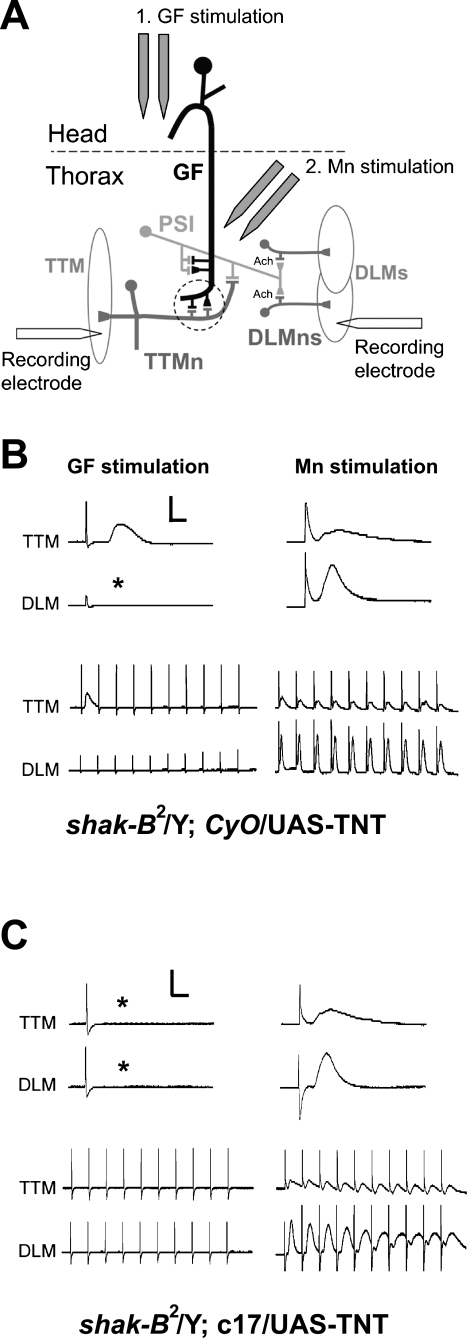
NMJ function is unaffected by the *shak-B*^2^ mutation or expression of tetanus toxin using the c17 line. (A) Schematic showing the positions of the stimulating and recording electrodes for either GF or motorneuron stimulation. (B) Responses in TTM and DLM to a single stimulus, or ten stimuli at 250 Hz, in the brain (GF stimulation) or the thorax (Mn stimulation) from a *shak-B*^2^/Y UAS-TNT/*CyO* fly. (C) Responses in TTM and DLM to a single stimulus, or ten stimuli at 250 Hz, in the brain (GF stimulation) or the thorax (Mn stimulation) from a *shak-B*^2^/Y c17/UAS-TNT fly. In (B) and (C) Mn stimulation always resulted in a muscle response to every stimulus.

Typically, *shak-B*^2^ mutant flies show a long latency response in TTM that is very labile. This may be due to the chemical component of the GF-TTMn synapse that develops, in the absence of gap junctions. To test this hypothesis we used the GAL4-UAS system to selectively block chemical transmission from the GF to the TTMn. We targeted the expression of either an active form of the light chain of the tetanus toxin (TNT), or an inactive form of the toxin (IMPTNT), to the GF using the GF-specific P[GAL4] line c17 and UAS transgenes encoding the two forms of the toxin (Sweeney *et al*., 1995; Allen *et al*., 1999; Trimarchi *et al*., 1999). Hemizygous *shak-B*^2^ males that expressed TNT in their GFs gave no responses in TTM or DLM upon stimulation (Table 1; Fig. 2G–I). This suggests that the chemical component of the GF-TTMn synapse is responsible for the long latency response in TTM. Interestingly, these flies showed an increase in spontaneous activity in both TTM and DLM (see following traces, Fig. 2H and I). Other genotypes served as controls. Control female flies, either heterozygous for *shak-B*^2^ alone or heterozygous for *shak-B*^2^ and expressing IMPTNT, showed wild-type electrophysiological recordings upon activation of the GFs (Table 1; Fig. 2A–C). Females, heterozygous for *shak-B*^2^, expressing TNT in their GFs also exhibited wild-type responses (Table 1); presumably because the gap junctions between the GF and TTMn are sufficient for normal connectivity. Finally, *shak-B*^2^ hemizygous males, and *shak-B*^2^ hemizygous males expressing IMPTNT, showed the previously characterized mutant responses of a long latency and poor following to trains of stimuli for TTM and no responses in the DLMs (Baird *et al*., 1990; Table 1; Fig. 2D–F). To confirm that any long latency to TTM was due to a defective GF-TTMn synapse, the stimulating electrodes were placed into the thoracic ganglia of two of the *shak-B*^2^ hemizygous males, five of the *shak-B*^2^ c17/UAS-IMPTNT males and five of the *shak-B*2 *CyO*/UAS-TNT males tested, to activate the motor neurons directly. This by-passes the GF and always resulted in short latency responses in both TTM and DLM, even if the fly had given no responses on GF stimulation (Fig. 3B and data not shown). To ensure that expression of the active tetanus light chain toxin was not affecting the neuromuscular junctions (NMJs) of either TTM or DLM, thoracic ganglia stimulation was also performed. Of the seven *shak-B*^2^ c17/UAS-TNT males, thoracic ganglia stimulation was performed on six and these all showed responses in both TTM and DLM (Fig. 3C and data not shown).

**Table 1:** Synaptic function in *shak-B*^2^ mutant flies expressing the tetanus light chain

		TTM			DLM		
			
Genotype	*n*	Latency (ms) *±* SEM	Following at 100 Hz *±* SEM	Following at 250 Hz *±* SEM	Latency (ms) *±* SEM	Following at 100 Hz *±* SEM	Following at 250 Hz *±* SEM
*shak-B*^*2*^/+	6	0.85 ± 0.03	100 ± 0.0%	100 ± 0.0%	1.43 ± 0.07	84 ± 10.7%	27.6 ± 7.2%
*shak-B*^*2*^/Y	7^a^	1.62 ± 0.17**	17.5 ± 5.5%	10.5 ± 0.5%	No responses	No responses†	No responses
*shak-B*^2^*/+; c17/IMPTNT*	7	0.87 ± 0.02	100 *±* 0.0%	88 *±* 9.2%	1.44 *±* 0.08	82.9 *±* 12.1%	33.1 *±* 8.2%
*shak-B*^*2*^/Y; c17/IMPTNT	6^b^	1.27 ± 0.1*	13.5 ± 1.7%	10.5 ± 0.5%	No responses	No responses	No responses
*shak-B*^2^*/+; c17/TNT*	12	0.92 *±* 0.02	97.7 *±* 1.3%	78.7 *±* 7.6%	1.49 *±* 0.04	92.7 *±* 5.0%	43.5 *±* 8.6%
*shak-B*^2^*/*Y; c17/TNT	7	No responses	No responses	No responses	No responses	No responses†	No responses†
*shak-B*^2^/+; CyO/TNT	7	0.86 ± 0.03	100 ± 0.0%	86.3 ± 6.4%	1.38 ± 0.06	91.1 ± 8.9%	40.6 ± 9.4%
*shak-B*^2^*/Y*; *CyO/TNT*	7^c^	1.36 *±* 0.09**	18 *±* 7.3%	12 *±* 2.5%	No responses	No responses	No responses

a 3/7

b 2/6

c 2/7 flies gave no responses in both TTM and DLM. TTM averages are from those that did respond.

† Occasionally PSPs were recorded but were spontaneous muscle contractions and not responses to the stimuli.

** *P* < 0.001

* *P* < 0.005 in a Student's unpaired *t*-test compared to *shak-B*^2^/+ females.

Statistical analysis of the results (see legend to Table 1) suggests that the chemical component of the synapse is responsible for the long latency response seen in TTM and this is blocked by expression of tetanus toxin. As tetanus toxin blocks chemical transmission generally the results do not provide any information concerning the transmitter.

### Identifying the GF as a cholinergic neuron

**Fig. 4:**
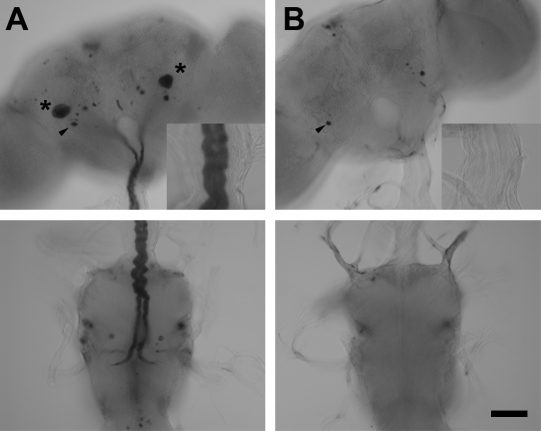
The GF is a cholinergic neuron. Dissected adult nervous systems stained for LacZ. (A) UAS-lacZ; A307 control preparation showing distinct staining in the GFs (*) and a few other cells in the brain and ventral nerve cord including a cell that lies just ventral to each GF (arrowhead). Inset is a higher power view of a cervical connective through which the labelled GFs can be easily identified. (B) UAS-lacZ; A307; *cha^3.3kb^*-GAL80 preparation. Note the lack of staining in the GFs but the presence of staining in the small ventral cell (arrowhead) that is in the position to be a cell body of a giant commissural interneuron. Inset higher power view of a cervical connective shows the GFs to be present but unlabelled. Scale bar, 50 µm; 25 µm for insets.

The major excitatory neurotransmitter in the *Drosophila* CNS is ACh (Lee & O'Dowd, 1999). Previous studies, using antibodies against choline acetyltransferase (ChAT) or generating a cha-GAL4 line, have shown extensive expression in the adult CNS but not identified the GF as cholinergic (Gorczyca & Hall, 1987; Yasuyama *et al*., 1996; Salvaterra & Kitamoto, 2001). We examined the CNS from cha-GAL4 flies expressing GFP carefully, but were not able to identify the GF as a cholinergic neuron unequivocally as the large domains of expression made such identification problematic (data not shown). To determine whether the GF is cholinergic we utilized a *cha*^*3.3kb*^-GAL80 line (Kitamoto, 2002) and reasoned that if the GF was cholinergic, expression of GAL80 protein in the neuron would inhibit GAL4-mediated expression of a UAS-reporter. When flies were generated containing the GAL4 line A307 that expresses in the GFs, a UAS-lacZ reporter transgene and *cha*^*3.3kb*^-GAL80, the reporter could not be detected in the GFs in any of the preparations (*n* = 15), even with excessive staining, but could in other GAL4-expressing neurons (Fig. 4). This was also the case for a second GF-expressing GAL4 line, c17 (data not shown). Controls showed 100% of the GFs examined to stain (*n* = 9). This indicates that the GF is a cholinergic neuron.

### Reducing ACh using the cha^ts2^ mutant allele

**Fig. 5:**
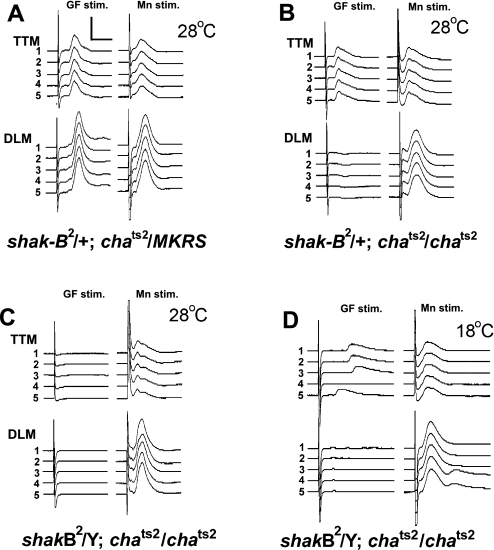
The response in the TTM is blocked in *shak-B*^2^; *cha*^ts2^ double-mutants at the restrictive temperature. Traces from the TTM and DLM of individual flies given five stimuli (1–5) at 1 Hz. (A) *shak-B*^2^/+ *cha*^ts2^/*MKRS* control female showing WT responses upon GF stimulation (GF stim) at 28 °C. (B) *shak-B*2/+ *cha*^ts2^/*cha*^ts2^ female showing a normal response in TTM and a loss of the DLM response at 28 °C. (C) *shak-B*^2^/Y; *cha*^ts2^/*cha*^ts2^ male showing no responses in DLM and a loss of responses in TTM at 28 °C. (D) *shak*-*B*^2^/Y; *cha*^ts2^/*cha*^ts2^ male showing no responses in DLM but responses in TTM at 18 °C. In all cases, individuals showed responses in both muscles upon thoracic stimulation (Mn stim.).Vertical scale bar, 50 mV; horizontal scale bar, 2 ms.

To test whether chemical synaptic transmission from GF to TTMn is cholinergic, we took advantage of a temperature sensitive allele of the *Drosophila cha* gene, which encodes choline acetyltransferase (ChAT), a major enzyme in ACh synthesis. We recorded responses to GF-activating stimuli in flies in which we had used a temperature sensitive allele of *cha* to reduce the amount of ACh within the CNS. The *cha*^ts2^ mutants are viable at 18 °C, but they die at the restrictive temperature of 30 °C due to severely reduced ChAT activity (Salvaterra & McCaman, 1985; Takagawa & Salvaterra, 1996). The protein produced from this allele is thermolabile, but the *cha* mRNA levels are also reduced in homozygous mutants after 48 h at 30 °C, which further reduces ChAT activity (Wang *et al*., 1999). Twenty-eight degrees C is considered a semipermissive temperature and adults shifted from 18 °C to 28 °C become paralysed but will move their legs if agitated. Females heterozygous for *shak-B*^2^ but homozygous for the *cha*^ts2^ allele and shifted to 28 °C exhibited normal responses to GF stimulation in TTM but no responses in DLM due to failure of the PSI-DLMns synapses (Table 2, Fig. 5B). This is consistent with the study of Gorczyca & Hall (1984) in which they used temperature-sensitive alleles of *cha* to determine that these peripheral synapses were cholinergic. *cha*^ts2^ females that were not temperature-shifted showed normal responses in both TTM and DLM (Table 2). This shows that there is sufficient ChAT activity in these flies for normal PSI-DLMns transmission even though protein levels are known to be somewhat reduced at the permissive temperature (Takagawa & Salvaterra, 1996). Females heterozygous for *shak-B*^2^ and *cha*^ts2^ that were reared at 18 °C, or shifted to 28 °C for 48 h, all showed normal responses upon GF stimulation (Table 2, Fig. 5A) indicating that the shift in temperature did not adversely affect synaptic function.

**Table 2:** Responses of *shak-B*^2^ and *cha*^ts2^ flies at 18 °C and 28 °C

			TTM *±* SEM		DLM *±* SEM	
				
Genotype	*n*	Temperature	GF stimulation	Mn stimulation	GF stimulation	Mn stimulation
*shak-B*^2^/+; *cha*^ts2^/*MKRS*	6	28 °C	100 *±* 0.0%	100 *±* 0.0%	100 *±* 0.0%	100 *±* 0.0%
*shak-B*^2^/+; *cha*^ts2^/*MKRS*	6	18 °C	100 *±* 0.0%	100 *±* 0.0%	100 *±* 0.0%	100 *±* 0.0%
*shak-B*^2^/+; *cha*^ts2^/*cha*^ts2^	6	28 °C	100 *±* 0.0%	100 *±* 0.0%	No responses	100 *±* 0.0%
*shak-B*^2^/+; *cha*^ts2^/*cha*^ts2^	6	18 °C	100 *±* 0.0%	100 *±* 0.0%	100 *±* 0.0%	100 *±* 0.0%
*shak-B*^2^/Y; *cha*^ts2^/*cha*^ts2^	6	28 °C	No responses	100 *±* 0.0%	No responses	100 *±* 0.0%
*shak-B*^2^/Y; *cha*^ts2^/*cha*^ts2^	6^a^	18 °C	27 *±* 13.2%	100 *±* 0.0%	No responses	100 *±* 0.0%
*shak-B*^2^/Y; *cha*^ts2^/*MKRS*	6^b^	28 °C	67 *±* 16.0%	100 *±* 0.0%	No responses	100 *±* 0.0%
*shak-B*^2^/Y; *cha*^ts2^/*MKRS*	6^c^	18 °C	77 *±* 16.7%	100 *±* 0.0%	No responses	100 *±* 0.0%

a 3/6

b 1/6

c 1/6 flies gave no responses in both TTM and DLM.

The percentage responses are calculated from all (6×5) stimuli given. At 28 °C, *shak-B*^2^/Y; *cha*^ts2^/*cha*^ts2^ flies gave significantly fewer responses than all other genotypes (Kruskal–Wallis anova, *H* = 14.94, *d.f.* = 3, *P* < 0.01). At 18 °C, *shak-B*^2^/Y; *cha*^ts2^/*cha*^ts2^ flies gave some responses but significantly fewer than all other genotypes (Kruskal–Wallis anova, *H* = 11.68, *d.f.* = 3, *P* < 0.01).

Hemizygous *shak-B*^2^ males that were also homozygous for *cha*^ts2^ and had been shifted to 28 °C gave no responses in DLM upon GF stimulation, as expected, but also gave no responses in TTM (Table 2, Fig. 5C). The chemical component of the GF-TTMn synapse is therefore not functional when ACh is reduced within the CNS. Of the six males of the same genotype, continually reared at 18 °C, three gave no responses and three gave characteristic long latency, intermittent, responses in TTM upon GF stimulation with a total of eight of 30 stimuli (27%) eliciting responses across the six preparations (Table 2, Fig. 5D). Function was decreased compared to controls (Table 2) indicating reduced ChaT activity of *cha*^ts2^ homozygotes at the permissive temperature. This is consistent with data reported by Salvaterra & McCaman (1985). *Shak-B*^2^; *cha*^ts2^/*MKRS* males also showed responses in TTM at 18 °C and 28 °C indicating that the temperature shift alone did not reduce synaptic function.

To confirm that the glutamatergic NMJs were unaffected by any reduction in ACh or change in temperature, we again used thoracic ganglia stimulation to activate TTMn and the DLMns directly. This resulted in responses in DLM and TTM irrespective of temperature or whether flies were homozygous for *shak-B*^2^ or *cha*^ts2^ (Mn stim, Fig. 5). Thus, the abolition of DLM responses in control flies, or TTM responses in *shak-B*^2^ flies, was due to failure of synapses within the CNS and not the NMJs.

## Discussion

We have used *shak-B*^2^ mutant flies to investigate the chemical component of the mixed GF-TTMn synapse within the CNS of *Drosophila*. By blocking chemical transmission in *shak-B*^2^ mutant flies using tetanus toxin we can deduce that the chemical component is functional in the absence of gap junctions. We have shown elsewhere that the GF is the only pathway from the brain to the TTMn (Allen *et al*., 2000) and yet when we remove the gap junctions a residual, albeit less reliable, pathway exists. Simultaneous removal of the gap junctions and blockade of cholinergic synapses in *shak-B*^2^; *cha*^ts2^ double-mutants blocks the GF-TTMn synapse at the restrictive temperature. When GAL80 is expressed under the control of a fragment from the *cha* promoter it blocks GAL4-mediated expression of a reporter in GFs. These results indicate that the chemical component of the GF-TTMn synapse uses ACh as its neurotransmitter.

Although the GFS is the most studied adult neural circuit in *Drosophila*, there are several elements of this escape pathway's outputs that are poorly understood. For example, the GF also activates the tibial levator (TLM; Trimarchi & Schneiderman, 1993), the dorsal ventral flight muscles (DVMs; Tanouye & Wyman, 1980), and possibly wing elevators (Tanouye & King, 1983; Hammond & O'Shea, 2007) but the neurons involved in this are unknown. As our analysis involves stimulating the GF and recording outputs to TTM and a DLM, the formal possibility still exists that there is a second parallel, unidentified, polysynaptic pathway from the GF to the TTMn that is uncovered when gap junctions are removed from the GF in *shak-B*^2^ flies. This is unlikely, however, as several studies in which the GF-TTMn presynaptic terminal has been perturbed exhibit a range of longer response latencies, corresponding with the morphological abnormalities seen (Allen *et al*., 1999; Allen *et al*., 2000; Godenschwege *et al*., 2002a; Godenschwege *et al*., 2002b; Godenschwege *et al*., 2006). This is consistent with a monosynaptic connection being weakened rather than ‘switching’ to a polysynaptic pathway. In addition, *shak-B*^2^ flies sometimes give no responses in TTM upon GF stimulation. If a second pathway existed, it would have to also have to have elements sensitive to loss of gap junctions formed by Shak-B. Our interpretation therefore explains the data best. Confirmation of GF-TTMn being monosynaptic only will require intracellular recordings from TTMn.

Several neural circuits have been identified in *Drosophila* that use mixed electro-chemical synapses including the GFS (Blagburn *et al*., 1999), sensory afferents from the halteres to flight motorneurons (Trimarchi & Murphey, 1997) and auditory pathways in the Johnston's organ (Sivan-Loukianova & Eberl, 2005). The results for the haltere afferents-to-B1 nicely parallel our results for the GF-TTMn. Both the haltere afferents onto the B1 motorneuron (Trimarchi & Murphey, 1997) and the GF-TTMn synapse (this study) are reduced in efficacy in *shak-B*^2^ mutant animals and the residual response is blocked by cholinergic blockers. Thus both mixed synapses use ACh as the transmitter and both contain gap junctions that require Shak-B. Given the range of behavioural phenotypes altered in *shak-B*^2^ mutants, it is unlikely that these will be the only synapses in the CNS that have these properties.

Electron microscopy and cell biological approaches have shown that the GF-PSI synapse is also a mixed electro-chemical synapse. Unlike GF-TTMn, it appears that the chemical component of this synapse is unable to function on its own as no responses are seen in *shak-B*^2^ mutants (Thomas & Wyman, 1984; Baird *et al*., 1990; this study). Although not demonstrated here, the chemical component of the GF-PSI mixed synapse is likely cholinergic. A recent study has determined that the Dalpha7 subunit of the nicotinic acetylcholine receptor (nAChR) is needed for transmission from the PSI to the DLMns and inputs to the GFs (Fayyazuddin *et al*., 2006). From the expression data of Fayyazuddin *et al*. this subunit seems not to be present at the GF-PSI or GF-TTMn synapses, however, this is yet to be determined.

It appears that by changing the properties of the GF during development the connectivity diagram was altered. We observed increased spontaneous activity in both TTM and DLM in *shak-B*^2^ mutant flies that were expressing TNT in the GFs throughout development. In contrast, we saw no spontaneous activity in *shak-B*^2^ *cha*^ts2^ males in which the GF-TTMn was blocked acutely in adults. Blocking chemical and electrical components of the GF may alter the homeostasis of TTMn and PSI early in development so that they receive greater input from other (thoracic) inputs. Blocking either of the components individually, or reduction of either component, has no noticeable effect. This suggests that chemical transmission from the GF to TTMn has a role during normal synaptic development. This dual role for the chemical and electrical components is not unprecedented as transient gap junction communication is needed for the correct development of chemical synapses in the optic lamina (Curtin *et al*., 2002). Indeed, activity is now seen as a vital aspect of neural cell development (Spitzer, 2006).

One developmental question that remains unanswered is whether the chemical component of the GF-TTMn synapse is stronger in *shak-B*^2^ flies than it is in wild type. It may make a stronger chemical synapse during development because there are no gap junctions present. The study of Blagburn *et al*. (1999) is inconclusive as to whether there are a greater number of chemical synaptic zones in *shak-B*^2^ flies compared to wild type. This hypothesis could be tested physiologically by recording from the motor neurons as performed by Fayyazuddin *et al*. (2006), or, genetically requiring either dominant negative expression or temperature sensitive mutations of *shak-B* to acutely block gap junctions.

Now that we have a better understanding of this mixed synapse and can dissect the two components genetically, we can analyse further the development and plasticity of the synapse. Studies of the role of activity can take advantage of these data to determine whether neural activity affects the development of the synapse. And studies of plasticity of the synapse can be combined with blockade of activity to assess the normal development of this synapse. Such analyses should shed light on the development and function of all mixed synapses.

## Abbreviations

ACh: acetylcholine
ChAT: choline acetyltransferase
DLMns: dorsal longitudinal muscle motorneurons
DLMs: dorsal longitudinal muscles
GF: giant fibre
GFS: giant fibre system
NMJ: neuromuscular junction
PSI: peripherally synapsing interneuron
TNT: tetanus toxin light chain
TTM: tergotrochanteral muscle
TTMn: tergotrochanteral motorneuron.
